# Perspectives of individuals with sickle cell disease on barriers to care

**DOI:** 10.1371/journal.pone.0265342

**Published:** 2022-03-23

**Authors:** Shannon Phillips, Yumei Chen, Rita Masese, Laurence Noisette, Kasey Jordan, Sara Jacobs, Lewis L. Hsu, Cathy L. Melvin, Marsha Treadwell, Nirmish Shah, Paula Tanabe, Julie Kanter

**Affiliations:** 1 College of Nursing, Medical University of South Carolina, Charleston, SC, United States of America; 2 Department of Hematology/Oncology, UCSF Benioff Children’s Hospital Oakland, Oakland, CA, United States of America; 3 School of Nursing, Duke University, Durham, NC, United States of America; 4 Department of Pediatrics, Medical University of South Carolina, Charleston, SC, United States of America; 5 Translational Health Research Division, RTI International, Research Triangle Park, NC, United States of America; 6 Department of Pediatrics, Comprehensive Sickle Cell Center, University of Illinois at Chicago, Chicago, IL, United States of America; 7 Department of Public Health Sciences, Medical University of South Carolina, Charleston, SC, United States of America; 8 Department of Pediatrics/Division of Hematology, UCSF Benioff Children’s Hospital Oakland, Oakland, CA, United States of America; 9 School of Medicine, Duke University, Durham, NC, United States of America; 10 Division of Hematology & Oncology, University of Alabama at Birmingham, Birmingham, AL, United States of America; Center for Primary Care and Public Health, SWITZERLAND

## Abstract

**Introduction:**

Sickle cell disease (SCD) is an inherited hemoglobinopathy that predominantly affects African Americans in the United States. The disease is associated with complications leading to high healthcare utilization rates, including emergency department (ED) visits and hospitalizations. Optimal SCD care requires a multidisciplinary approach involving SCD specialists to ensure preventive care, minimize complications and prevent unnecessary ED visits and hospitalizations. However, most individuals with SCD receive sub-optimal care or are unaffiliated with care (have not seen an SCD specialist). We aimed to identify barriers to care from the perspective of individuals with SCD in a multi-state sample.

**Methods:**

We performed a multiple methods study consisting of surveys and interviews in three comprehensive SCD centers from March to June 2018. Interviews were transcribed and coded, exploring themes around barriers to care. Survey questions on the specific themes identified in the interviews were analyzed using summary statistics.

**Results:**

We administered surveys to 208 individuals and conducted 44 in-depth interviews. Barriers to care were identified and classified according to ecological level (i.e., individual, family/interpersonal, provider, and socio-environmental/organizational level). Individual-level barriers included lack of knowledge in self-management and disease severity. Family/interpersonal level barriers were inadequate caregiver support and competing life demands. Provider level barriers were limited provider knowledge, provider inexperience, poor provider-patient relationship, being treated differently, and the provider’s lack of appreciation of the patient’s SCD knowledge. Socio-environmental/organizational level barriers included limited transportation, lack of insurance, administrative barriers, poor care coordination, and reduced access to care due to limited clinic availability, services provided or clinic refusal to provide SCD care.

**Conclusion:**

Participants reported several multilevel barriers to SCD care. Strategies tailored towards reducing these barriers are warranted. Our findings may also inform interventions aiming to locate and link unaffiliated individuals to care.

## Introduction

Sickle cell disease (SCD) is an inherited hemoglobinopathy that affects approximately 100,000 individuals in the U.S., over 90% of whom are African American or Black [1]. SCD is characterized by alterations in the shape of red blood cells with subsequent blood vessel occlusion, infarction, organ damage, and inflammation [2]; these sequelae may cause significant pain, the hallmark symptom of SCD. Pain often begins in childhood, with progressive organ damage and disease-related complications occurring throughout the lifespan, contributing to declining quality of life [3,4]. Individuals with SCD have high acute care utilization rates, including ED visits, hospitalization, and rehospitalization [5]. However, during late adolescence and young adulthood, when individuals with SCD typically transition from pediatric to adult care, remarkable increases occur in SCD complications, risk of death, emergency department (ED) use, hospitalization, and rehospitalization [5–9]. Concurrently, rates of outpatient clinic visits decrease [8]. Ongoing, comprehensive care delivered by a multidisciplinary team with SCD expertise is critical to minimize complications and associated increases in ED and hospital utilization [10,11]. A key barrier to ongoing care is access to adult SCD specialists, which is influenced by difficulty navigating the adult healthcare system, transportation barriers, and lack of adult SCD specialists [12,13]. In addition to not receiving ongoing comprehensive SCD care, individuals without a designated SCD specialist, termed “unaffiliated patients,” often do not receive necessary disease education or the opportunity to participate in research or advocacy.

Identifying individuals with SCD who are unaffiliated is challenging in the U.S. because there is no national registry which limits patient tracking procedures for those who are not engaged in the healthcare system. Typically, information about unaffiliated patients is only obtained from experienced clinicians, community groups, patient advocates, and word of mouth. Understanding the barriers to access to SCD-specific care for these individuals is critical to developing strategies to locate and engage unaffiliated patients in care [14]. Previous research on barriers to care for individuals with SCD in the US has been conducted from the perspectives of adults with SCD, adolescents with SCD, caregivers of children with SCD, healthcare providers, and stakeholders (e.g., individuals in community-based organizations). These studies focused on barriers to adherence to the treatment regimen [15], barriers to and interventions for improving treatment [16], barriers to primary care [17], barriers to clinic attendance [18], barriers to care transition [19], barriers to care in Northern California [20], challenges and facilitators to caring for individuals with SCD in the ED in North Carolina [21], and barriers to receiving care in the ED [22]. Prior studies primarily focused on narrow aspects of care (e.g., barriers to appointment adherence) and were conducted at single sites; further, only one used multiple or mixed methods approaches. Importantly, these studies did not specifically focus on barriers to receiving SCD-specific care. A multi-site study of broad, overarching barriers to SCD-specific care using multiple or mixed methods is lacking and may provide a high-level understanding of barriers to care from the perspective of individuals with SCD. Thus, the purpose of this study is to explore the perspectives of individuals with SCD in a multi-state sample to develop a comprehensive understanding of the ecological barriers to establishing and maintaining SCD care.

## Methods

### SCDIC overview

The Sickle Cell Disease Implementation Consortium (SCDIC) was established in 2016 to improve access to healthcare and implementation of the National Heart, Lung and Blood Institute (NHLBI) recommended SCD care guidelines [23]. The SCDIC consists of eight comprehensive SCD centers across the United States and one data coordinating center [23].

In Phase I of the SCDIC program, sites conducted a community-based needs assessment (NA) utilizing a multiple-methods approach [24] with cross-sectional surveys, focus groups, and key informant interviews with individuals with SCD and providers. The data collection instruments for the NA were developed by the SCDIC NA working group, with representation from each site [25]. The working group also identified additional measures that sites could electively choose from for an “enhanced” survey and interview protocol exploring barriers to care. Only three SCDIC sites [two located in the Southeast (SE) and one in the West (W)] administered the “enhanced” survey and interview along with the NA survey at their respective locations. This study analyzes the data collected from the enhanced survey and interview protocol.

### Setting

All three institutions are academic health science centers with specialty outpatient services dedicated to the care of individuals with SCD and serve urban- and rural-dwelling individuals. Site 1, located in the SE, provides pediatric, adult, and transition-specific care for individuals with SCD. Providers include adult and pediatric sickle cell specialists and advanced practice providers. Multidisciplinary support consists of psychology, social work, a transfusion specialist, a SCD educator, and genetic counseling services. Acute management services are provided through a dedicated sickle cell day hospital. Site 2, located in the W, is primarily a pediatric facility; however, it does provide lifespan sickle cell specialty care on an outpatient basis as well as within a day hospital that provides infusion and transfusion services. Staff and healthcare personnel include pediatric and adult sickle cell specialists, advanced practice providers, a transfusion specialist, psychology and social work support, and a patient navigator. Once patients are over the age of 22 years (corresponding with the transition of a state-specific insurance), inpatient admissions and subspecialty care occur in community facilities. At the time of the study, SCD services at Site 3 (also located in the SE) included a lifespan SCD outpatient clinic that served individuals with SCD of all ages, access to other subspecialty providers for referral, and a sickle cell infusion clinic for transfusion and pain management. Staff and healthcare providers consisted of adult and pediatric hematologists/oncologists and sickle cell specialists, advanced practice providers, a dedicated psychologist, care coordinators, and social worker support.

### Participant inclusion/exclusion criteria

Individuals were eligible for participation if they first, had a self-reported SCD diagnosis; access to the electronic health record for confirmation of the diagnosis was not included in IRB approval to minimize participant risk (confirmation of diagnosis occurred prior to participant involvement at the location of recruitment). Second, participants were required to live in the geographic region of one of the three participating sites for the enhanced survey. Third, participants were required to be between 15–50 years of age; the age range was set by the funding agency to focus on access to healthcare in the age range during which marked increases are observed in mortality, chronic pain, organ damage, and acute care utilization. Lastly, participants were required to not be experiencing acute symptoms of SCD at the time of survey or interview to minimize participant burden when unwell or in pain. Participants were recruited through multiple venues (i.e., clinician referrals, website posts, recruitment flyers and letters, health fairs and medical conferences, and clinical programs). Ethical approval was obtained from the Institutional Review Boards at the three SCDIC sites prior to data collection. Adult participants signed informed consent, and the legal guardian signed the consent on behalf of a minor participant, who gave assent. Participants were compensated with gift cards.

### Data collection

#### Interviews

The development of the semi-structured interview guide was informed by prior literature and additional input from sickle cell experts in all sites [26,27]. The guide included open-ended questions and probes related to personal experiences with SCD care, access to and communication with healthcare providers, transition from pediatric to adult care, and urgent needs (interview guide in S1 File). Focus groups or individual interviews took place in private rooms in outpatient settings where participants typically received care, or by phone. The interviewers were study coordinators or principal investigators trained in qualitative methods. Interviews were audio-recorded, lasted approximately 45–75 minutes, and were transcribed and redacted to remove confidential information.

#### Surveys

The 46-item “Access Barriers Checklist: Advocates” instrument, a checklist of barriers to care developed at Oregon Health & Sciences University [27] was adapted for this study. SCDIC experts later added specific SCD-related items [26]. The final checklist consisted of 53 items and was organized into 8 categories: transportation, access to services, insurance, provider knowledge and attitudes, accommodations and accessibility, social support, and individual and SCD-specific barriers. The checklist was scored by summing the number of barriers checked. The total score for the SCD Barriers to Care Checklist ranges from 0 to 53 and has demonstrated face validity and test-retest reliability (Pearson *r* = 0.74, *p* < 0.05) [26].

The barriers to care checklist was administered in person, on a tablet, or using paper and pencil. A study coordinator remained nearby to answer questions, provide clarification or troubleshoot any technical difficulties. For individuals with known or observed difficulties with reading, the instruments were administered verbally by a study coordinator. Participants were also offered the option to complete the survey by phone. All study coordinators were trained and monitored in the administration of the surveys.

### Quantitative analysis

Survey data were entered and stored electronically in a REDCap [28,29] database hosted by the SCDIC data-coordinating center. Data analyses were conducted using SAS statistical software version 9.04. Descriptive statistics were presented as means, frequencies, and percentages where appropriate.

### Qualitative analysis

Data analysis was conducted using a deductive-inductive approach. A deductive codebook was developed by the SCDIC based on existing literature and perceived common underlying themes in previous interviews. Using this codebook, the three participating centers coded one transcript and compared coding results. Coders held weekly meetings to share coding progress and discuss potential changes to the codebook (e.g., removing redundant or unrepresentative codes, adding inductive/emerging codes that were not included in the initial coding scheme). Coders collectively determined the number of themes and subthemes identified in the interviews. Additionally, the numbers of interview participants contributing to each theme or subtheme were tallied to determine the frequency representation of each theme and subtheme. To measure the extent to which coders at each site assigned the same segments in the sample transcript to the same code, percentage agreement was calculated. We had an overall 62.5% agreement with 74% of segments agreed upon by 3 or more of the 5 coders. NVivo qualitative data analysis software was used to code, organize, and manage data. We analyzed sections of each transcript in which barriers to care were discussed. As coding progressed, coders regularly convened to resolve disagreements and refine the codebook. Each of the general themes in the codebook was further divided into sub-codes as the study team developed a better understanding of the complexities of the data. Using both iterative and constant comparative analytic methods [27], we adopted a reflexive approach to better understand the data. The study adhered to the consolidated criteria for reporting qualitative studies (COREQ) guidelines (COREQ checklist).

### Triangulation

Interview and survey data were triangulated using methodological triangulation for completeness to increase understanding of the phenomenon being examined [30]. In this case, data were triangulated to develop a comprehensive understanding of barriers to care as perceived by individuals with SCD. Triangulated data was organized into themes and subthemes and categorized by ecological level [31].

## Results

Demographic and clinical characteristics of survey and interview participants are presented in Table 1. Surveys were completed by 208 adolescents and adults with SCD; the majority characteristics included 58.2% female, 44.2% aged 19–30 years, 95% Black or African American, and 90.4% non-Hispanic or Latino. Among the 44 interview participants, the mean age was 31.1 years, 97.8% were African American or Black, non-Hispanic or Latino, and 54.5% were female. Barriers to care were identified in 8 themes and 10 subthemes on the following ecological levels: socio-environmental or organizational; provider; family/interpersonal; and individual. Survey results are presented in Table 2. Frequencies and illustrative quotes are presented in Table 3.

**Table 1:** Participant demographics.

Site Characteristics	1	2	3	Cumulative
**Interview Sample (n)**	14	15	15	44
**Age**				
Mean (SD)	32.5 (5.7)	29.7 (9.3)	30.0 (7.4)	31.1 (7.8)
Median	32	N/A	30	N/A
Minimum-Maximum	25–43	15–46	17–44	15–46
Missing, n	1	0	N/A	1
**Ethnicity, n (%)**				
Non-Hispanic/ Black	13 (93.0)	15 (100.0)	15 (100.0)	43 (97.8)
Hispanic/ Black	1 (7.0)	0	0	1 (2.2)
**Gender, n (%)**				
Male	5 (36.0)	9 (60.0)	6 (40.0)	20 (45.5)
Female	9 (64.0)	6 (40.0)	9 (60.0)	24 (54.5)
**Insurance, n (%)**				
Private insurance only	2 (14.3)	1 (6.7)	1 (6.7)	4 (9.1)
Medicare only	2 (14.3)	2 (13.3)	N/A	4 (9.1)
Medicaid only	4 (28.6)	N/A	5 (33.3)	9 (20.5)
State Sponsored Health Plan	0	3 (20.0)	2 (13.3)	5 (11.4)
Private Insurance + Medicare	1 (7.1)	N/A	N/A	1 (2.2)
Medicare+ Medicaid	3 (21.4)	9 (60.0)	6 (40.0)	18 (40.1)
Missing, n	2	N/A	N/A	2 (4.5)
**Survey Sample (n)**	52	58	98	208
**Age, n (%)**				
≤18	2 (3.8)	7 (12.1)	8 (8.2)	17 (8.2)
19–30	24 (46.2)	22 (37.9)	46 (46.9)	92 (44.2)
31–50	17 (32.7)	29 (50.0)	36 (36.7)	82 (39.4)
>50	2 (3.8)	0	0	2 (1.0)
Missing	7 (13.5)	0	7 (8.2)	15 (7.2%)
**Gender, n (%)**				
Male	26 (50.0)	25 (43.1)	33 (33.7)	84 (40.4)
Female	24 (46.2)	33 (56.9)	64 (65.3)	121 (58.2)
Missing	2 (3.8)	0	1 (1.0)	3 (1.4)
**Race, n (%)**				
Black/African American	49 (94.2)	54 (93.1)	95 (96.9)	198 (95.0)
White	0	0	0	0
Other	0	3 (5.2)	1 (1.0)	4 (2.0)
Missing	3 (5.8)	1 (1.7)	2 (2.0)	6 (2.9%)
**Ethnicity, n (%)**				
Non-Hispanic or Latino	45 (86.5)	51 (87.9)	92 (93.9)	188 (90.4)
Hispanic or Latino	3 (5.8)	5 (8.6)	0	8 (3.8)
Missing	4 (7.7)	2 (3.4)	6 (6.1)	12 (5.8)
**Highest Degree Received, n (%)**				
Less than High School	4 (7.7)	8 (13.8)	20 (20.4)	32 (15.4)
High school graduate/GED equivalent	10 (19.2)	13 (22.4)	22 (22.4)	45 (21.6)
Some college	16 (30.8)	13 (22.4)	33 (33.7)	62 (29.8)
College graduate or professional	16 (30.8)	27 (46.6)	20 (20.4)	59 (28.4)
Missing	6 (11.5)	1 (1.7)	3 (3.1)	10 (4.8)
**Employment, n (%)**				
Working	13 (25.0)	20 (34.5)	18 (18.4)	51 (24.5)
Disabled	17 (32.7)	18 (31.0)	37 (37.8)	72 (34.6)
Student	8 (15.4)	7 (12.1)	15 (15.3)	30 (14.4)
Unemployed- Looking for work	3 (5.8)	6 (10.3%)	16 (16.3)	25 (12.0)
Other	8 (15.4)	7 (12.1)	7 (7.1)	22 (10.6)
Missing	3 (5.8)	0	5 (5.1)	8 (3.8)
**Marital Status, n (%)**				
Married	7 (13.5)	7 (12.1)	10 (10.2)	24 (11.5)
Not married but living together	6 (11.5)	3 (5.2)	16 (16.3)	25 (12.0)
Widowed, divorced, annualled or separated, not living together	2 (3.8)	7 (12.1)	6 (6.1)	15 (7.2)
Never been married	35 (67.3)	41 (70.7)	59 (60.2)	135 (64.9)
Missing	2 (3.8)	0	7 (7.1)	9 (4.3)
**Income, n (%)**				
$20,000 and under	17 (32.7)	39 (67.2)	60 (61.2)	116 (55.8)
$20,001 - $50,000	13 (25.0)	9 (15.5)	19 (19.4)	41(19.7)
$50,001+	8 (15.4)	8 (13.8)	6 (6.1)	22 (10.6)
Missing	14 (26.9)	2 (3.4)	13 (13.3)	29 (13.9)
**Insurance, n (%)**				
Private insurance only	12 (23.1)	13 (22.4)	8 (8.2)	33 (15.9)
Medicare or Medicaid	33 (63.5)	45 (77.6)	77 (78.6)	155 (74.5)
Other	2 (3.8)	0	3 (3.1)	5 (2.4)
No coverage	0	0	1 (1.0)	1 (0.5)
Missing	5 (9.6)	0	9 (9.2)	14 (6.7)
**Sickle Cell Phenotype, n (%)**				
SS or Beta 0	39 (75.0)	41 (70.7)	70 (71.4)	150 (72.1)
SC Disease	8 (15.4)	12 (20.7)	22 (22.4)	42 (20.2)
Other variants	3 (5.8)	2 (3.4)	2 (2.0)	7 (3.4)
Missing	2 (3.8)	3 (5.2)	4 (4.1)	9 (4.3)

**Table 2:** Survey results^a^.

Question	Cumulative N (%)	Site 1 N (%)	Site 2 N (%)	Site 3 N (%)
**1. Transportation Barriers**				
No barriers	119 (61.7)	33 (68.8)	31 (56.4)	55 (61.1)
At least 1 barrier	74 (38.3)	15 (31.3)	24 (43.6)	35 (38.9)
I can’t get transportation	16 (21.6)	2 (13.3)	3 (12.5)	11 (31.4)
Public transit is not easy to get to	22 (29.7)	6 (40.0)	9 (37.5)	7 (20.0)
Transportation costs too much for me	19 (25.7)	3 (20.0)	8 (33.3)	8 (22.9)
I do not have a vehicle	45 (60.8)	8 (53.3)	12 (50.0)	25 (71.4)
I do not have access to a vehicle	7 (9.5)	1 (6.7)	0 (0.0)	6 (17.1)
Sample Size	193	48	55	90
**2. Access to Services**				
No barriers	165 (85.5)	44 (91.7)	44 (80.0)	77 (85.6)
At least 1 barrier	28 (14.5)	4 (8.3)	11 (20.0)	13 (14.4)
I don’t know where to get care	15 (53.6)	2 (50.0)	5 (45.5)	8 (61.5)
I can’t get care because the health care providers’ office is too far away	12 (42.9)	2 (50.0)	7 (63.6)	3 (23.1)
Sample Size	193	48	55	90
**3. Insurance Barriers**				
No barriers	126 (65.3)	36 (75.0)	30 (54.5)	60 (66.7)
At least 1 barrier	67 (34.7)	12 (25.0)	25 (45.5)	30 (33.3)
My insurance does not cover the services I need	19 (28.4)	3 (25.0)	10 (40.0)	6 (20.0)
My insurance will not let me go where I want to get services	15 (22.4)	2 (16.7)	9 (36.0)	4 (13.3)
My insurance does not cover services that will keep me well	12 (17.9)	3 (25.0)	5 (20.0)	4 (13.3)
My insurance does not cover medicines, or my co-pay are too high	26 (38.8)	3 (25.0)	14 (56.0)	9 (30.0)^a^
My insurance does not cover services that allow communication between different providers, which might lead to less informed decisions	12 (17.9)	1 (8.3)	6 (24.0)	5 (16.7)
Health care services are too expensive because of the co-pay or share of cost	17 (25.4)	3 (25.0)	6 (24.0)	8 (26.7)
It takes too long to get approval for the care that I need	20 (29.9)	2 (16.7)	9 (36.0)	9 (30.0)
My insurance paperwork is too hard to fill out	7 (10.4)	2 (16.7)	4 (16.0)	1 (3.3)
Getting reimbursement for some treatments or services is hard	9 (13.4)	3 (25.0)	2 (8.0)	4 (13.3)
My insurance will not cover needed services if I have to go to a different county	11 (16.4)	3 (25.0)	4 (16.0)	4 (13.3)
Sample Size	193	48	55	90
**4. Provider Knowledge and Attitudes**				
No barriers	85 (44.0)	21 (43.8)	20 (36.4)	44 (48.9)
At least 1 barrier	108 (56.0)	27 (56.3)	35 (63.6)	46 (51.1)
Providers don’t believe that I have genuine pain and need help	54 (50.0)	13 (48.1)	20 (57.1)	21 (45.7)
I am not seen quickly enough when I am in pain	72 (66.7)	16 (59.3)	27 (77.1)	29 (63.0)
Providers accuse me of drug-seeking	52 (48.1)	13 (48.1)	22 (62.9)	17 (37.0)
Providers let me know that they do not appreciate how knowledgeable I am about my disease	23 (21.3)	3 (11.1)	9 (25.7)	11 (23.9)
It is hard for me to find a provider who has enough experiences with or knowledge about sickle cell disease	49 (45.4)	10 (37.0)	23 (65.7)	16 (34.8)
I am treated differently from other patients	36 (33.3)	10 (37.0)	12 (34.3)	14 (30.4)
Communication between me and the providers has been difficult	43 (39.8)	11 (40.7)	11 (31.4)	21 (45.7)
Sample Size	193	48	55	90
**5. Health Care Facilities Access and Accommodation**				
No barriers	123 (64.7)	31 (66.0)	26 (47.3)	66 (75.0)
At least 1 barrier	67 (35.3)	16 (34.0)	29 (52.7)	22 (25.0)
Places for me to go to learn how to stay well are not close by or easy to get to	38 (56.7)	7 (43.8)	18 (62.1)	13 (59.1)
The health care providers’ hours are not convenient for me	16 (23.9)	7 (43.8)	7 (24.1)	2 (9.1)
The wait in the health care office is too long for me	29 (43.3)	12 (75.0)	8 (27.6)	9 (40.9)
The paperwork I have to fill out is too much	10 (14.9)	2 (12.5)	5 (17.2)	3 (13.6)
I could not get an appointment	14 (20.9)	2 (12.5)	8 (27.6)	4 (18.2)
Sample Size	190	47	55	88
**6. Social, Family, and Caregiver Support**				
No barriers	118 (61.5)	33 (70.2)	28 (50.9)	57 (63.3)
At least 1 barrier	74 (38.5)	14 (29.8)	27 (49.1)	33 (36.7)
I do not have enough support	23 (31.1)	2 (14.3)	11 (40.7)	10 (30.3)
The people who take care of me or give me support are burned out	28 (37.8)	7 (50.0)	13 (48.1)	8 (24.2)
I am burned out by taking care of others or by giving support to them	16 (21.6)	4 (28.6)	4 (14.8)	8 (24.2)
I need help with daily chores/ just doing daily activities	33 (44.6)	7 (50.0)	12 (44.4)	14 (42.4)
I am socially isolated	23 (31.1)	5 (35.7)	8 (29.6)	10 (30.3)
There are other things going on in my family that are more important than my health care	11 (14.9)	4 (28.6)	4 (14.8)	3 (9.1)
It is hard to make appointments because it is hard for me to find childcare	12 (16.2)	2 (14.3)	5 (18.5)	5 (15.2)
Sample Size	192	47	55	90
**7. Barriers for Individuals**				
No barriers	126 (67.0)	34 (72.3)	27 (50.0)	65 (74.7)
At least 1 barrier	62 (33.0)	13 (27.7)	27 (50.0)	22 (25.3)
I don’t really know what to do to stay healthy	17 (27.4)	3 (23.1)	8 (29.6)	6 (27.3)
I don’t know enough about the sickle cell disease care that I need	7 (11.3)	2 (15.4)	2 (7.4)	3 (13.6)
I don’t understand the system or find it too hard to work through	17 (27.4)	5 (38.5)	7 (25.9)	5 (22.7)
It is hard to follow up on care (for example, by going to the pharmacy, taking medicines at the right time, or making follow up appointments)	16 (25.8)	2 (15.4)	11 (40.7)	3 (13.6)
I miss appointments because of memory problems	16 (25.8)	3 (23.1)	7 (25.9)	6 (27.3)
Staff are hard to talk to	13 (21.0)	5 (38.5)	5 (18.5)	3 (13.6)
Staff are hard to understand	9 (14.5)	3 (23.1)	1 (3.7)	5 (22.7)
The medical system is very confusing to me	14 (22.6)	4 (30.8)	6 (22.2)	4 (18.2)
I have too many different health problems, so it is hard for me to make sickle cell disease care a priority	6 (9.7)	2 (15.4)	3 (11.1)	1 (4.5)
It is hard for the staff to get a hold of me (for example, I move a lot or don’t have a phone)	6 (9.7)	0 (0.0)	4 (14.8)	2 (9.1)
Sample Size	188	47	54	87
**8. Barriers Related to Sickle Cell Disease**				
No barriers	34 (17.7)	10 (20.8)	8 (14.5)	16 (18.0)
At least 1 barrier	158 (82.3)	38 (79.2)	47 (85.5)	73 (82.0)
Worry or fear	89 (56.3)	21 (55.3)	31 (66.0)	37 (50.7)
Frustration or anger	90 (57.0)	22 (57.9)	28 (59.6)	40 (54.8)
Lack of confidence	44 (27.8)	10 (26.3)	15 (31.9)	19 (26.0)
It is hard to be assertive	34 (21.5)	6 (15.8)	11 (23.4)	17 (23.3)
It is embarrassing	31 (19.6)	7 (18.4)	11 (23.4)	13 (17.8)
I am concerned about the costs	26 (16.5)	6 (15.8)	8 (17.0)	12 (16.4)
I am tired	117 (74.1)	26 (68.4)	37 (78.7)	54 (74.0)
I am in pain	123 (77.8)	28 (73.7)	36 (76.6)	59 (80.8)
Sample Size	192	48	55	89

^a^ Not all participants responded to every item, therefore, sample size is reported for each domain and percentages are reported relative to the subgroups.

**Table 3:** Themes, frequencies, and illustrative quotes.

Theme or Subtheme	Frequency	Illustrative Quotes
**Socio-environmental/organizational Level**		
Insurance	31	• “The co-pays, it gets to be quite difficult especially whenever you have as many appointments as we have. I mean we may have as many as three to four appointments a month” (Part. 9) • “I know the doctor has said if you start to feel bad what you can do is double up on the morphine. So, I go, and I give them a prescription and they give me a months’ worth of morphine. So, if it’s the 18^th^ and I’m starting to feel bad and I need to double up, then I’m going to run out before the next one comes, and the insurance companies won’t fill it until it’s the 30^th^ again unless I’m in the hospital.” (Part. 26) • “My medical expenses are very high at times and I’m on my family’s insurance plans, so if our insurance isn’t covering, or is choosing not to cover something then it’ll get like sent to collections and then we’ll get lots of calls about it. And that’s definitely something that we’re trying to figure out how to handle because there’s not very much advocacy, in terms of like teaching people how to handle it or handling it for them.” (Part. 37)
Transportation	30	• “Well, I don’t have a car, so the only real barrier is the distance from here to [the clinic]. My mother has to drive me, and we have to really plan that because of her schedule and stuff so that’s really the only barrier.” (Part. 35) • “When you don’t have your own transportation because relying on people for rides is a headache or if you don’t have money to get on the bus it’s a headache. So, it’s like when you have doctor’s appointments and you can’t get there, it’s like you got a bad face because you didn’t come to your appointments.” (Part.12)
Systems Roadblocks and Administrative Barriers	24	• “I think one of the biggest challenges is that the more frequent appointments that I have, I don’t get calls for those. So, I have to try to, you know, remember to keep up with those and the times. I have requested to have the calls; I don’t get it. The MyChart system is a good system, but I’m not able to get into it and I’ve told people about it, but I still haven’t received any help for it.” (Part. 13) • “The only drawback was being able to reach out if I was going through a crisis. It was kinda hard to contact them (the clinic). When you call it was more of an answering machine, and a lot of times if that was a Friday, you wouldn’t get a call back of course until Monday, and by that time the crisis is about over.” (Part. 29) • “There’s a bunch of going and beating around the bush and not being able to schedule an appointment in a range where you can see a doctor in at least a day, the next day, or even the day after that. I know some people have to schedule appointments, but they won’t be able to get an appointment for two or three weeks out.” (Part. 33)
Access to Care: Clinic Availability	16	• “I really didn’t have a steady doctor because it’s so hard to find a doctor who will treat sickle cell patients after they turn 18.” (Part. 5) • “I’ve called several (doctors) and one retired and another left the area and then another never returned my call so, and I called several times and didn’t get anything back, so I don’t really know what happened but with the other things going on I kind of you know, that just kind of stalled.” (Part. 9) • “I was able to kind of find one (doctor) that accepted my Medicaid cause otherwise a lot of times some of those places don’t accept certain Medicaid.” (Part. 25)
Access to Care: Poor Care Coordination	15	• “I wish I had one doctor for everything like instead of going and seeing one doctor for this and one doctor for that and they all tell me different things.” (Part. 12) • “The primary doctor doesn’t communicate to the sickle cell doctor and it’s hard because sometimes it’s through emails and sometimes it’s lost in translation. It would be nice if sometimes they could have joint clinics, joint clinics for the primary care doctor and the hematologist specialist doctor to see the patient at the same time and they can network and talk through what problem they’re having.” (Part. 39)
Access to Care: Service Limitations	12	• “He (the doctor) was okay. He was more about getting the pain under control at that moment and not the long-term solution for it, so it was just a bunch of medicine taking with him instead of trying to get to the actual solution.” (Part. 27) • “I think the only differences (when seeing a only a pediatrician and not a hematologist) was not being able to come and get pain management, I wasn’t able to do that. I had to go to the emergency room whenever I was in pain.” (Part. 24) • “The emphasis is most definitely on medication…your main thing is this, don’t forget you have to get your prescription, you have to come get your blood transfusions, like–and I also can’t really talk to you about holistic care cause that’s not my specialty.” (Part. 37)
Access to Care: Provider/Clinic Refusal	11	• “He was a wonderful doctor; however, he wanted to concentrate more on his oncology patients. He was slowly getting rid of his sickle cell patients. He felt like he could not treat me to the level in which I needed to be treated with his sickle cell patients.” (Part. 5) • “If you live in a city where they don’t have a clinic that specializes in sickle cell and you’re just trying to find a hematologist, I found that they are hesitant to take on sickle cell patients. They’ll typically say no you need to see this specific hematologist, go to this specific institution, this specific clinic. And I’ve even had one clinic go so far as to tell me the reason that they do that is because they don’t want to manage the medication.” (Part. 36)
**Provider Level**		
Provider Inexperience and Lack of Training	23	• “Knowing about sickle cell and knowing how to treat it isn’t a very common thing, surprisingly. When you go to a place that doesn’t have great sickle cell care, you’re not going to be seen in the same way that you would be seen at a place that does.” (Part. 37) • “I don’t have a primary care physician. I’ve been looking for one, but I think the biggest barrier is trying to speak with the primary care doctor well enough to see if they know enough about sickle cell that they can actually be my primary care physician.” (Part. 13) • “If I go to like a primary care doctor, she barely understood what sickle cell was and all that and not understand, like, if I needed pain medicine, she was very hesitant to give it to me and it’s very hard.” (Part. 34)
Provider-Patient Relationship	23	• “Ever since I’ve switched to adult hematology, I have yet to establish like any type of close relationships with the doctors. Really, essentially, you treat may care, you know, and you manage my medication, and monitor my levels to make sure I’m not going too far in either direction. And that’s pretty much been it.” (Part. 36) • “You can tell when a person is very passionate about something and when they’re not and they just didn’t seem like they were passionate about it. It was just like, “Well, here. Take this. Here, take that.” “What am I taking it for?” They couldn’t even tell me that.” (Part. 9) • “I felt like they didn’t really understand sickle cell. They probably just got a class on it when they were in college, but the way they were acting, it felt like they didn’t really understand it, know it, probably never had a family member with it to know what it’s like. So, it felt like it was a new relationship every time I went in. It wasn’t like I’ve been seeing this patient for a year and a half.” (Part. 25)
Lack of Appreciation of SCD Knowledge	19	• “They (healthcare providers) should be trained better and let the patient have a say-so because the patient knows what he needs…This is what I need. I know it. I’ve been through this 100 times. I know.” (Part. 21) • “I can’t stand doctors who have this idea and they’re not going to listen to the patient. No matter what you say, they’re going to do what they’re going to do. We know what’s going on with our bodies. We know how long it takes for us–I mean I can’t stand it when a doctor tells me, “oh, a sickle cell crisis lasts this long and then it’s over.” I’m like, it’s not every patient is the same. I know that.” (Part. 35) • “The only thing I think that don’t work is when the doctors think they know more–they think they know more about me than I do. Not even being that they know more about sickle cell than me, but they think they know more about me than I know about myself. That’s the only thing that really irritates me.” (Part. 41)
Lack of Trust	17	• “I know it’s chronic pain, but I know my body and it’s worse when something’s wrong. And some people just don’t listen. So, it makes me frustrated and angry.” (Part. 2) • “My relationship (with a previous provider) wasn’t as good as it is here. I felt like I couldn’t really talk to them without them judging me. I felt like they really didn’t understand sickle cell.” (Part. 4) • “My issue is more of a trust factor. I don’t like doctors who just assume…I don’t want nobody putting me, just because I have sickle cell, in the same bracket as people with sickle cell. I mean, I know I have sickle cell; I’m aware I have sickle cell, but we’re all different and we all, you know, respond differently.” (Part. 11)
Treated Differently	14	• “They claim that I missed 3 appointments and so they had to release me. I felt like that was false. I actually felt like the hematologist also works for cancer patients…And I felt like, I’m just going to be honest, they make more money off the people with cancer than sickle cell people because we mostly have Medicaid.” (Part. 4) • “The stigmatism that haunts sickle cell patients about the opioids and morphine. You know, that’s a hot topic now all over the United States but what it really is, is just really people’s personal opinions. You see, our society accepts and treats cancer patients differently. They can’t see that sickle cell disease is something you’re born with. I’d say their pain is less than ours. However, they get better treatment, pain management than sickle cell patients.” (Part. 5) • “How come people with sickle cell dying quicker than people with cancer? You don’t want none of them to die, but ask a lot of people, “Do you know what sickle cell is?” and they be like, “What is that?” We can’t even get a little commercial on the TV. It hurts, in more than one way. It’s like, dang, they don’t know nothing about sickle cell at all.” (Part. 9)
**Family/Interpersonal Level**		
Social, Family, and Caregiver Support: Overwhelmed Supports	13	• “I do manage it, but sometimes it’s very difficult to manage it on my own. Sometimes after going to the ER and you’re admitted into the hospital and discharged after two to three weeks of being in the hospital, you go home, and you don’t have the energy to do anything. And coupled for some of us that live alone and don’t have family around, though even if you do have family, they still have to go to work or responsibilities to take care of not you and you alone. It’s so hard to get around and do stuff that you need to do, like laundry, sometimes even taking a shower or making a quick meal for yourself and all that.” (Part. 39) • “I don’t really have friends and family. I just don’t. They don’t support. The only come to me when they need something.” (Part. 2) • “It was just me and my daughter. You can’t take them with you, I mean you can, but it’s like, not the ideal thing to do because for one, it distracts you off of maybe some of the questions you were going to come in and ask because you’re making sure your child is not tearing up the place.” (Part. 25)
Social Family and Caregiver Support: Competing Life Demands	11	• “So much is going on and I just remember, you know, my appointment’s coming up, I need to schedule it. It may seem easy to go ahead and schedule an appointment, but it’s not always that easy.” (Part. 4) • “Once I’ve been in the emergency room, I don’t want to come to the doctor’s office and there’s other things I have to do. People with sickle cell have a life. I have a daughter, a job, school, so it’s kind of like, do I want to take this two hours to go up here to tell her (the provider) that I went to the emergency room or do I want to take this two hours to do something else?” (Part. 13)
**Individual Level**		
Disease-Specific Barriers	16	• “Pain, and fatigue, tiredness, and I thought that I was going to be able to make it to Wednesday. That was my transfusion day, but every day started feeling like an eternity. I don’t think I could wait that long. Wednesday is kid of a long time, and the last time I decided to wait, the pain probably actually forced me to go to the ER.” (Part. 21) • “If I’ve missed an appointment, it’s because of like being sick or something. Not necessarily because of not being able to physically get there.” (Part. 23) • “I know people are getting diagnosed, and they’re handed a pamphlet about sickle cell disease, and they’re kind of left to navigate this complex disease on their own.” (Part. 44)
Lack of Knowledge in Self-Management	4	• “When I first got on the medicine, I wasn’t necessarily taking it how I should’ve been. I was young at that age, and I was going through a lot of insecurities about the whole sickle cell and stuff.” (Part. 7) • “I have a late onset, so I didn’t grow up with sickle cell. I had sickle cell in me, but I didn’t get any of the symptoms until I was like 28 and so a lot of the knowledge that everybody has gathered through their years, I don’t have. So, I think when I see someone and they know I have sickle cell, they assume my level of knowledge is about the same or my experience is the same as others but it’s totally different.” (Part. 43)

### Socio-environmental and organizational level

#### Insurance

Challenges associated with insurance were: high co-pays; limitations in coverage of providers, medications, and services; extended length of time for approval; complex processes and paperwork; and issues specific to disability. Approximately 1/3 of survey participants across sites endorsed insurance barriers; site-specific results ranged from 25% (Site 1) to 45.5% (Site 2). The most commonly reported barrier was inadequate coverage for medicines and high copays. Correspondingly, interview participants described challenges finding providers or clinics who accept their insurance and accrued debt as a result of limitations in coverage or multiple co-pays. Gaps in coverage of medications, specifically, the expense of co-pays for medications (particularly with multiple medications to fill per month), running out of pain medications (insurance covered a 30-day supply), and having too many prescriptions per month (insurance only covers a certain number per month). At times, these gaps in coverage and inability to obtain pain medication led to emergency department (ED) visits and/or hospitalizations.

#### Transportation

Transportation barriers included lack of reliable transportation, long distance to the clinic or provider, public or insurance-funded transportation, transportation expenses, and difficulties with finding transportation while feeling poorly or taking medications for pain. Transportation was indicated as a barrier by 38.3% of survey participants across sites, with site-specific proportions ranging from 31.1% at Site 1 to 43.6% at Site 2. The most commonly reported barrier was not having a personal vehicle (60.8%). According to interview participants, lack of reliable transportation included not having a personal vehicle and relying on others for help, which sometimes led to missed appointments. Living an extended distance from the clinic limited access to care; participants described as much as a three-hour drive one way for appointments. Long distances compounded transportation issues with greater planning necessary to arrange transportation with family or friends, traffic considerations, higher gas costs, and coping with pain during a long ride. Participants who relied on insurance-funded transportation described waiting at the clinic until all patients were finished with appointments and setting up transportation with a three-day advance notice, which was at times difficult to remember and led to missed appointments.

#### Systems roadblocks or administrative barriers

Barriers pertained to challenges with conveniences of healthcare services, such as obtaining appointments promptly, inconvenient clinic hours, complex procedures and paperwork, and difficulties contacting providers. Barriers in this theme were reported by 35.3% of survey participants across sites, ranging from 25% (Site 3) to 52.7% (Site 2). The most commonly endorsed barriers were not having a place to go to stay well nearby (56.7%) and too long a wait at clinics (43.3%). Accordingly, interview participants reported waits for appointments at specialists’ offices for as long as two years. Participants also described a desire to schedule appointments in a shorter amount of time following an ED visit or hospitalization or if feeling an urgent need to be seen. Participants reported challenges with getting appointments at day hospitals or IV therapy clinics for pain management, often because of limited space and shared services with oncology patients. Further, participants explained difficulties with getting in contact with providers, particularly when feeling poorly or experiencing a crisis, and with delays in responses, particularly over weekends. Clinic hours and procedures presented another barrier; changes in clinic hours led to difficulties with arranging work schedules for appointments, and clinic rules regarding which services are offered on certain days added complexity to making appointments.

#### Access to care

Access to care barriers were infrequently reported by survey participants across sites (14.5%) and ranged from 8.3% at Site 1 to 20% at Site 2. Despite the low number of survey participants reporting access barriers, access barriers were highly reported by interview participants and included barriers not captured via survey. The most frequently endorsed barrier on surveys was not knowing where to get care (53.6%), which was reflected in discussions of clinic availability with interview participants. Participants described being unable to find an adult provider (with SCD expertise), particularly after leaving pediatric care or when the current provider moved. They also reported a lack of providers or specialists in a rural area or other location. In one case, a participant had an adult SCD provider, but the clinic closed for financial reasons leading to difficulties finding a new provider with similar knowledge.

Poor care coordination, specifically, provider-to-provider and provider-to-patient communication and inconsistency in care presented barriers to care access. Participants described being told different and conflicting messages from multiple providers, which was stressful and confusing. Participants also described situations where coordination between providers was perceived to be insufficient and reported that the PCP and SCD specialists communicate, but not as much as they would like, and a desire to speak with the PCP and SCD specialist concurrently to discuss problems. Another participant described having the onus of information transfer, as the PCP was unable to access records from the SCD specialist.

Service limitations barriers reflected a lack of sickle cell care components, such as pain management, prescription of disease-modifying therapy, or lab services. In some cases, participants were seen by a provider who only did lab work and did not prescribe medications. Conversely, some were seen by a provider who only prescribed pain mediation and did not do lab work or provide disease-specific education and guidance. Participants also described provider hesitance to prescribe pain medications and limitations in prescribing practices. One participant mentioned feeling as though the provider or clinic emphasized medical care and treatments and not holistic care, such as yoga and other complementary approaches.

While some participants described barriers to finding care, others described barriers related to provider/clinic refusal, which included situations in which the clinic provider declined to continue to provide SCD care. A few participants described being dismissed from practice by a hematologist/oncologist who also saw individuals with cancer because either the provider no longer wanted to care for individuals with SCD or did not feel comfortable caring for individuals with SCD.

### Provider level

#### Provider knowledge and attitudes/provider characteristics

Across sites, 56% of survey participants reported barriers to care pertaining to provider knowledge and attitudes; site-specific proportions ranged from 51.1% (Site 3) to 63.6% (Site 2). A commonly reported barrier in this theme pertained to provider inexperience and lack of training in the care of SCD, which affected participants’ perceptions of the care they received and influenced their ability to find a provider with whom they felt comfortable. Overall, gaps in SCD knowledge included a general lack of education and understanding of SCD care, resulting in participants encountering providers who don’t know what to do or how to handle SCD management and exacerbation. Nearly half (45.7%) of survey participants reported difficulties with finding a provider who is knowledgeable in SCD as a barrier to care; site-specific proportions of participants endorsing this barrier ranged from 34.8% (Site 3) to 65.7% (Site 2). Examples described by interview participants included having a provider who only prescribed pain medications without understanding SCD pathophysiology or SCD-specific treatments, leading to participants finding another provider with a greater understanding of SCD. One participant described the providers at the SCD clinic as “stagnant” and subsequently stated their hopes that new providers in the clinic will introduce new ideas and treatments for SCD management.

Key provider-patient relationship barriers included a lack of a close patient-provider relationship, perceived lack of provider empathy and understanding, desire for a better rapport with providers, and gaps in patient-provider communication. Difficulties with patient-provider communication was reported as a barrier by 39.8% of participants across sites and ranged from 31.4% (Site 2) to 45.7% (Site 3). In a few cases, interview participants experienced impersonal relationships and dissatisfaction with current or past providers, stating that clinic visits felt as though they were on “autopilot”—each visit was like the first time with the provider (even though they saw the same provider every time). Similarly, some participants described a lack of comfort with providers, particularly when seeing someone new or when feeling a lack of empathy from the provider. In other cases, participants mentioned losing a long-standing, personal relationship when transitioning from pediatric to adult care or transitioning to a new provider after their provider left the practice. Gaps in provider-patient communication led to relationship barriers, such as feeling as though the provider doesn’t listen to patients.

Providers’ lack of appreciation of SCD knowledge of individuals about their condition and participant perceptions of having little or no shared decision-making in their treatment plan were important barriers on the provider level. Approximately 21% of survey participants across sites reported this barrier, ranging from 11.1% at Site 1 to 25.7% at Site 2. Most frequently, interview participants described feeling as though providers don’t listen to them and a desire for providers to better understand the patient’s feelings and experiences. Participants verbalized feeling as though their voice didn’t matter and frustration and anger when providers don’t listen to them. One participant elaborated on feelings of being uninvolved in decision-making and explained the importance of providers speaking with patients about their care instead of making generalizations and telling them what they need.

Lack of trust in the provider-patient relationship stemmed from participant perceptions that providers do not believe they are in pain and being accused of drug-seeking. Across sites, 48.1% of participants reported being accused as drug-seeking as a barrier to care, which ranged from 37% (Site 3) to 62.9% (Site 2). Terms used by interview participants to describe their experiences included feeling a “stigma of SCD as drug seekers,” being “lumped together” with all individuals with SCD, being “treated like a theme,” being “judged,” and being “stereotyped.” Participants also described being unable to find the right “fit” because of provider attitudes. In one case, a participant mentioned feeling as though there is a lack of providers who will “fight for” their patients.

Being treated differently from other patients who do not have SCD also contributed to barriers to care. Approximately 1/3 of survey participants across sites reported this barrier; proportions by site were comparable, ranging from 30.4% (Site 3) to 37% (Site 1). Interview participants frequently compared treatment of individuals with SCD to those with cancer and reported feeling as though patients with cancer receive better treatment and pain management, including prioritizing patients with cancer for IV therapy. A few participants described the role of societal perceptions of cancer versus SCD, stating that “society accepts and treats cancer patients differently” and noting that “many don’t know what SCD is, but everyone knows what cancer is.” Participants also described being treated differently more generally (as opposed to compared with cancer).

### Family/Interpersonal level

#### Social, family, and caregiver support

Participants reporting barriers related to overwhelmed supports described challenges with having no or inadequate support from family, friends, parents/caregivers, or the community, and support systems being “burned out.” Over 1/3 of survey participants (38.5%) reported barriers pertaining to social, family, and caregiver support; site-specific proportions ranged from 29.8% (Site 1) to 49.1% (Site 2). Survey participants most frequently endorsed a need for help with daily activities as a barrier to care (44.6%). During interviews, circumstances in which participants reported having inadequate support included assistance with healthcare decision making and post-hospitalization or post-ED visits when they are distraught or don’t have the energy for daily activities. In terms of community support, participants described being unaware of or unable to find SCD support groups. One participant explained SCD affecting people of color who often reside in communities that are not economically stable compounds the issue of lack of support.

Competing life demands barriers referred to perceptions that other life or family events are more important than healthcare. Most participants described conflicts between work, school, or childcare and healthcare appointments. Participants’ busy lives and multiple responsibilities made it difficult to remember appointments and carve out time for appointments.

### Individual level

Individual-level barriers included disease-specific barriers, which pertained to the effects of SCD that limit care-seeking behaviors, predominantly burdensome symptoms and emotions such as pain, fatigue, frustration, worry, and depression that contributed to not feeling well enough to attend appointments. Similarly, disease-specific barriers were the most highly endorsed barriers to care among survey participants across sites (82.3%), with site-specific proportions ranging from 79.2% (Site 1) to 85.5% (Site 2). Some interview participants described having a milder disease process without SCD pain or recalled a period when they experienced less severe disease, which led to not seeking care.

Other individual-level barriers were endorsed by 1/3 of survey participants across sites, ranging from 25.3% (Site 3) to 50% (Site 2). Survey participants reported lacking knowledge in how to stay healthy (27.4%) and not knowing enough about the SCD care they need (11.3%). Similarly, a perceived lack of knowledge in SCD self-management described by interview participants pertained to not knowing how to stay healthy or not understanding the care needed for SCD. All participants described a process of learning how to manage their care. Participants described being younger and having insecurities related to SCD with an associated lack of adherence to treatments and having a late onset of SCD effects, which meant not gathering needed knowledge about their health status.

## Discussion

Patients with SCD must navigate a challenging terrain of barriers to engaging in high-quality SCD-specific health care. Participants in this study reported various barriers at the socio-environmental/organizational-, provider-, family-, and individual-level. Some challenges experienced by participants in this study are similar to those previously documented in the scientific literature, such as system, financial, and communication barriers [32,33]. However, this study demonstrates a comprehensive perspective of overarching barriers to care and supports the relative importance of addressing barriers at the system and provider level. The aims of the current study are consistent with recommendations to address barriers using a multi-factorial approach [16]. Further, results add a translational perspective to previous studies exploring the impact of stigma [34] and racial discordance [35] on the care of patients with SCD.

Barriers on the socio-environmental and organizational levels were among the most prevalent in this study. Difficulty with transportation was a significant challenge, particularly for participants who lived in a community that did not have a sickle cell specialist. Among prior studies investigating barriers to care, transportation was infrequently mentioned by adolescents with SCD as a barrier to clinic attendance [18] but was more frequently endorsed as a barrier to care among adolescents and adults with SCD in Northern California [20], suggesting adolescents rely on parents/caregivers for transportation and lack of reliable transportation is a more common barrier among adults with SCD. Insurance-funded transportation was an essential source of support for many participants, but its use still involved overcoming barriers that were not an issue with private transportation. In addition to transportation issues, diverse barriers pertaining to the structure of sickle cell clinics, such as difficulty contacting clinics, extended wait times, and inconvenient hours, indicated in previous literature [16,17,21,32,36] were also reported. Lack of care coordination and limited sickle cell specialty clinic availability were critical issues for many participants. Addressing these system-level barriers is especially important in light of previous evidence indicating the serious impact they have on healthcare seeking behavior [32]. This evidence supports previous literature highlighting the value of roles such as case managers [36] or community health workers (CHWs), who may be particularly adept at supporting health system navigation with respect to social and cultural aspects of the patient experience [37].

Individuals with SCD reported that access to an SCD specialist (a provider-level barrier) was a key barrier, consistent with prior studies on barriers to care for individuals with SCD [16,19,20]. As a patient perspective, this finding stands out in contrast to a recent study reporting provider perspectives from emergency department physicians that patient behavior is the most important barrier to SCD care [21]. Participants reported an overall lack of trust, and poor relationships and communication with many healthcare providers. Additionally, participants reported providers often do not appear to have sufficient knowledge of SCD and do not respect the patient’s own experience and expertise with their body, particularly among providers who are not SCD experts. These findings further support results of a 2009 systematic review [16] and a 2015 study [19] in which the most consistently patient-reported barrier to SCD pain management was negative provider attitudes and provider lack of SCD knowledge. The fact that our findings are consistent with those of a systematic review published over a decade ago demonstrate the continued need to address these barriers. Shared decision-making is a potential area of intervention that has been suggested related to SCD care, including collaborative education [38] and decision aids such as written or visual materials [39]. Importantly, if socio-environmental/organizational level barriers are addressed, and patients can engage in sustained relationships with expert SCD providers, many of the provider-level barriers may be bypassed as patients can connect with experienced SCD professionals.

Family/interpersonal- and individual-level barriers were also reported, though these were not as prevalent as the barriers at the socio-environmental/organizational- and provider-levels. Participants reported social supports who were at times overwhelmed along with competing life demands, such as the care of children. Few prior studies on barriers to care identified lack of social support as a key barrier, though strained relationships and diminished family support were reported barriers to transition for individuals with SCD [19]. CHWs may also be an important source of support for patients who need interpersonal support, as they are themselves community members [40]. Participants described their stage of development and understanding as a barrier to SCD care at times, particularly when they struggled to understand care options adequately enough to engage in the decision-making process. High-quality communication, either given by or augmented by members of the patients’ racial or cultural group [33] may support patients to gain confidence in their decision-making skills.

All themes identified in this study included some perceptions of discordance between patients and the organizations, providers, and sometimes even communities with which they engaged. Researchers are increasingly recognizing the impact a variety of determinants of health–socioeconomic status, race and ethnicity, age–have on individuals’ health outcomes. Despite increased efforts to amend the disparities created by social determinants of health, minimal progress has been made in populations with SCD. To improve the quality of care and quality of life for people with SCD, researchers should adopt an intersectional approach to study the interconnectedness between minority status and chronic illness. Intersectionality is a way of understanding social representation in terms of how systems of race, social class, and gender overlap with no one category taking primacy [41]. Disparities in health outcomes for people with SCD are evident across racial and socioeconomic groups [42]. Research on a different population, African American mothers living with HIV, has shown that the larger the intersection, the more vulnerable the affected populations, and the more complex the process of accessing quality health care—leading to a greater likelihood of poor health outcomes [43]. Similarly, patients with SCD suffer from health-related stigma, such as being called a derogatory term “sickler” at the emergency department [44]. Negative attitudes from healthcare professionals and perceived stigma from the public can lead to delay in care and ineffective pain treatments. Research on stigma and health disparities in SCD populations could benefit from understanding how different aspects of individuals’ identities intersect [34], and apply that knowledge to design culturally appropriate and personalized interventions.

While the qualitative (interview) findings in this study were comparable across sites, site-specific differences in the quantitative (survey) findings were observed. For most themes, one site had higher proportions of participants endorsing barriers; however, the difference in proportions among sites for the majority of barriers was narrow. Differences among sites may be related to several factors. For instance, the availability of services and resources within the SCD clinic (or at the same location) varies by site whereby patients at one site may more easily and conveniently receive SCD care while at another site, services may need to be sought in the larger community. In addition, while all sites in this study serve urban and rural-dwelling patients, the geographic region served varies by site and may lead to transportation being a more significant barrier at one site over another. Finally, assessment of barriers to SCD care may have been conducted more consistently and regularly at one site over another, contributing to greater participant awareness of multilevel barriers to care rather than actual experience of greater barriers. Regardless, findings support the need to compare approaches across organizations serving individuals with SCD and to determine ways to provide more consistent, quality care across organizations. Initiatives such as the recently formed National Alliance for Sickle Cell Centers (NASCC) [45], which provides support and infrastructure to organizations to improve the quality of care for individuals with SCD, are key to improving quality, consistent care.

Our study has several limitations. The study was conducted in three academic SCD centers in the U.S. and may not represent other health facilities across the nation. Additionally, all our participants were affiliated with care; their perspectives may not reflect all individuals with SCD, limiting the generalizability of our findings. Another potential limitation of the study is the representativeness of the sample. Participant age was limited to 15–50 years; future studies should include all age groups for broader representation, including those older than 50 years who have been historically underrepresented in clinical trials and qualitative studies in SCD. In this study, interested individuals excluded due to age were invited to leave their contact information with research staff for future studies. Finally, qualitative studies have examined other barriers in SCD in more depth but with smaller numbers [46]. Despite these limitations, our study has several important strengths. Our multiple method study design enabled us to combine the richness of qualitative data with our survey findings. We were able to identify key themes that emerged from the 44 in-depth interviews and explore their generalizability across the broader survey respondents.

## Conclusion

Individuals with SCD encounter several multilevel barriers to SCD-specific care. These barriers are likely even greater for individuals unaffiliated from SCD care and may be a reason they have become unaffiliated. There is a clear need to develop strategies that address factors at individual, family, provider, and socio-environmental and organizational levels. Findings from this study will guide interventions tailored towards mitigating those barriers and increasing affiliation with SCD care.

## Supporting information

S1 File Interview guide. (DOCX)
