# Supplementary material for: Perspectives of individuals with sickle cell disease on barriers to care
Source: PLoS One. 2022 Mar 23;17(3):e0265342. doi: 10.1371/journal.pone.0265342 (PMC8942270; doi:10.1371/journal.pone.0265342)
Supplement: S1 File — (DOCX) [file pone.0265342.s001.docx]

**Sickle Cell Disease Implementation Consortium Needs Assessment:**

**Patients Focus Group and Individual Interview Guide**

**Session Information**

Interview date:

Interview team:

Participant ID number:

**Participants Information**

Gender (male/female/other):

Age:

Race (self-identify):

Ethnicity (self-identify):

Current education level:

Employment status:

Marital status:

Household income level:

Current health insurance coverage:

1. **Patient access to primary and specialty care**

*Intro statement for facilitator to read: Many individuals living with SCD often see many different type of healthcare providers. These could include your hematologist, sickle cell specialist, primary care doctor, nurse practitioners, specialists to take care of your kidneys, lungs, hips, etc., social workers, pain specialists care coordinators, and psychologists or therapists. We would like to understand what kind of providers you see and any barriers you experience related to this, or things that work really well to help make sure you can get all the healthcare you need.*

***Overarching question*: What kind of healthcare providers do you see? (or who do you see for your healthcare?) (Facilitator note: try to guide responses specific to type of provider they are discussing)**

*Probes:*

- Describe your relationship with these providers
- Are there any barriers you experience?
- Is insurance a barrier?
- Is transportation a barrier?
- Is the location of the clinic or health center a barrier?
- Any facilitators?
- Do you work with a care coordinator?
- Do you work with a social worker, therapist or psychologist?
- What works/doesn’t work?
- If you could change one thing about the care you receive, what would it be?
- “Have you had to miss appointments due to childcare?”

1. **Patient comfort level taking HU**

*Intro statement for facilitator to read:* *Hydroxyurea is a drug that is taken by many persons with sickle cell to help prevent pain crisis and other complications. We would like to ask you some questions about taking HU, whether or not you currently take it, have taken it in the past, or have thought about taking it.*

***Overarching question*: Tell us about HU, whether you use it, decided not to, or just what you have heard about?**

*Probes:*

- Do you take HU? (This question may be too direct may need an introduction)

If yes

Do you feel like you need education or more information on how to manage hydroxyurea dosing?

Why do you take HU? Or For what reasons are you prescribed HU? (To address health literacy, adherence)

How long have you been taking it?

What made you decide to start taking it?

What other factors did you consider to decide to start taking it?

Whose advice did you seek before deciding to take it?

Do you use any apps, props, or phone features to help you manage your HU? If yes, which ones?

Do you use any websites to help you with taking HU? If yes, which ones?

Are there any barriers to taking it daily?

Is insurance a barrier to getting your prescription filled?

F/u: If No

What made you decide not to take HU?

If your doctor recommended HU for you, what might change your mind to take it?

Have you ever heard about HU or had it prescribed?

Do you know anyone who takes HU?

If yes, what did you hear about HU from them?

1. **Beliefs and practices related to pain control**

*Intro statement for facilitator to read: Many patients with sickle cell experience both acute pain, often referred to as vaso-occlusive crisis or episodes, and/or chronic pain at some point in their lives. We would like to talk about that now.*

***Overarching question:* Tell me about the pain you experience and how you manage it.**

*Probes:*

- Tell me about medicines you take to manage your pain.
- Do you have problems with being given or picking up opiates? If yes, tell me more.
- What else do you do to manage pain other than meds?
- Do you have a pain plan? What is it?
- Is insurance a barrier to getting your prescriptions filled?

1. **Emergency Department**

*Intro statement for facilitator to read*: Many persons living with SCD have had to go to the emergency department for treatment of either pain or other complications. We would like you to discuss those experiences.

**Overarching question: Tell me about your experience in the emergency department?**

*Probes:*

- When and why did you decide to go?
- How was your pain managed?
- Was there any follow up recommended?
- Was there any difficulty in getting the recommended follow-up?
